# Impact of Na Concentration on the Phase Transition Behavior and H^−^ Conductivities in the Ba–Li–Na–H–O Oxyhydride System

**DOI:** 10.1002/advs.202203541

**Published:** 2022-11-16

**Authors:** Kei Okamoto, Fumitaka Takeiri, Yumiko Imai, Masao Yonemura, Takashi Saito, Kazutaka Ikeda, Toshiya Otomo, Takashi Kamiyama, Genki Kobayashi

**Affiliations:** ^1^ Solid State Chemistry Laboratory Cluster for Pioneering Research (CPR) RIKEN Wako 351–0198 Japan; ^2^ Department of Structural Molecular Science School of Physical Sciences SOKENDAI (The Graduate University for Advanced Studies) Okazaki 444–8585 Japan; ^3^ Department of Materials Molecular Science Institute for Molecular Science Okazaki 444–8585 Japan; ^4^ Japan Science and Technology Agency (JST) Precursory Research for Embryonic Science and Technology (PRESTO) 4‐1‐8 Honcho Kawaguchi Saitama 332‐0012 Japan; ^5^ Institute of Materials Structure Science High Energy Accelerator Research Organization (KEK) Ibaraki 305–0801 Japan; ^6^ Department of Materials Structure Science School of High Energy Accelerator Science SOKENDAI (The Graduate University for Advanced Studies) Ibaraki 305–0801 Japan

**Keywords:** hydride ion conductors, oxyhydrides, phase transition, solid electrolyte

## Abstract

K_2_NiF_4_‐type Ba–Li oxyhydride (BLHO) transitions to a so‐called hydride superionic conductor, exhibiting a high and essentially temperature‐independent hydride ion (H^−^) conductivity over 0.01 S cm^−1^ through the disordering of H^−^ vacancies above 300 °C. In this study, a Ba–Li–Na–H–O oxyhydride system synthesized in which lithium is partially substituted with sodium in BLHO and investigated the effects of Na content on the phase transition behavior and the conductivity. Structural refinements and differential scanning calorimetry experiments confirmed a lowering trend in the phase transition temperatures and decreasing enthalpy changes for the transition with increasing Na content. Substitution of not <40% of Li with Na lowered the degree of ordered vacancies at the H^−^ sites at room temperature and improved conductivities by more than two orders of magnitude in the low‐temperature region (*T* < 300 °C) before the phase transition. These findings clearly show that introducing Na into the lattice effectively stabilizes the high‐conductive phase of BLHO.

## Graphical abstract

K_2_NiF_4_‐type Ba–Li oxyhydride exhibits high hydride ion (H^−^) conductivity over 0.01 S cm^−1^ above 315 °C through a melt of ordered vacancies at hydride ion sites. In this work, a Ba–Li–Na–H–O oxyhydride system synthesized in which Li is partially substituted with Na. The introduction of Na reduced the degree of ordering of the H^–^ vacancies, resulting in a lower phase transition temperature and an increase in conductivity over two orders of magnitude in the low‐temperature range.

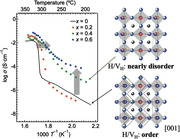

## 1 Introduction

Hydrogen transport in solids is crucial in electrochemical devices, such as fuel cells and batteries. The recent verification of hydride ion (H^−^) diffusion in solids^[^ ^1^ ^]^ and the demonstration of the potential battery reaction based on H^−^ conduction^[^ ^2^ ^]^ have led to the recognition of H^−^ as a new charge carrier of hydrogen. The low charge density (monovalence and moderate size) and high polarizability of H^−^ are advantageous for fast ion diffusion in solids, while its strong reducing ability (*E*°(H^−^/H_2_) = −2.23 V vs. SHE) should be adequate for high activity in energy (material) conversion and high energy density in batteries.

In the study on H^−^ conductors, mixed‐anion compounds such as oxyhydrides are currently the primary targets of materials exploration. This is because, compared to simple metal hydrides, the coexistence of multiple anions has facilitated the control of parameters essential for developing H^−^ conductors, such as the H^–^ concentration,^[^ ^2^, ^3^ ^]^ arrangement of H^−,[^ ^2^, ^4^ ^]^ and the bandgap.^[^ ^3^ ^]^ The method of constructing the H^−^ diffusion pathway by introducing hydride ions into the oxide framework structure or suppressing the electronic conduction of rare‐earth hydrides *Re*H_3_ (*Re* = Y, La) with doping of oxide ions has been mainly attempted. Outstanding materials have been reported, such as Ba_2−_ *_x_*_−_ *_y_* LiH_3−2_ *_x_* O_1−_ *_y_* (*x* = 0.15, *y* = 0.1),^[^ ^5^ ^]^ and LaH_3−2_ *_x_* O*_x_* (*x* = 0.25),^[^ ^3^ ^]^ which exhibit practical conductivities above 10^–2^ S cm^−1^ in the intermediate temperature range (300 < *T* < 400 °C). More recently, LaH_3−2_ *_x_* O*_x_* (*x* = 0.1) annealed under high hydrogen partial pressure has achieved H^−^ conduction around room temperature,^[^ ^6^ ^]^ denoting the progress of material development. However, applying H^−^ conduction to energy/material conversion requires solid electrolytes that can operate in a wide range from room temperature to intermediate temperature, and further progress in material development is needed. Here, we focus on Ba_2−_ *_x_*_−_ *_y_* LiH_3−2_ *_x_* O_1−_ *_y_* (hereafter called BLHO).

K_2_NiF_4_‐type BLHO has two stable compositions of nearly stoichiometric Ba_2_LiH_2.8_O_1.1_ and highly vacant Ba_1.75_LiH_2.7_O_0.9_. The former (*α*‐BLHO) is the high‐pressure phase and metastable composition under ambient pressure and has a simple K_2_NiF_4_‐type structure with space group *I*4/*mmm*. The latter one (*β*‐BLHO) is obtained by ambient‐pressure synthesis and forms the 2*a* × *b* × *c* superlattice structure with space group *Pnm*2_1_ at low temperatures due to three types of long‐range ordering, namely H_eq_/*V*_H_, H_ap_/O_ap_, and Ba/*V*_Ba_ (H_eq_, O_ap_, and *V_M_* represent hydrogen at equatorial anion sites, oxygen at apical anion sites, and a vacancy at *M* sites, respectively). These long‐range orders are successively lost upon heating, transforming to *γ*‐BLHO (space group: *Pnma*) at 300 °C, where only the H_ap_/O_ap_ ordering remains, and to *δ*‐BLHO (space group: *I*4/*mmm*) at 350 °C, where all long‐range orderings are disordered. The H^−^ conductivity enhances after the *β*‐*γ* transition to the so‐called superionic conduction state, which exhibits conductivity over 10^–2^ S cm^−1^ nearly independent of the temperature. In other words, if the superionic phase (*γ*‐ and *δ*‐BLHO) can be stabilized to a lower temperature, the operating temperature of BLHO as a solid electrolyte would expand. In this study, we attempted to stabilize the superionic phase by elemental substitution that has historically been used in many species of ionic conductors.^[^ ^7^ ^]^ Highly polarizable Na that is expected to be a suitable counter‐cation of H^−^ for fast diffusion^[^ ^8^ ^]^ was selected as the substituted element to Li. The crystal structure, phase transition behavior, H^−^ conductivity, and their correlation were investigated in a Ba–Li—Na—H–O solid solution system (hereafter called Na‐BLHO).

## 2 Results

### 2.1 Synthesis

**Figure 1:**
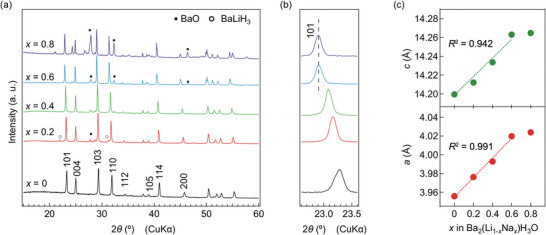
a) X‐ray diffraction patterns of Na‐BLHO synthesized by the high‐pressure reaction. b) Expanded XRD patterns of (101) reflection. c) Lattice constants estimated from Rietveld analyses to XRD data. The *R* ^2^ for the linear fits for *a*‐ and *c*‐axes are 0.991 and 0.942, respectively. Note that error bars are smaller than the symbols.

The Na‐BLHO, represented as nominal composition Ba_2_(Li_1−_ *_x_* Na*_x_*)H_3_O were synthesized by a high‐pressure reaction using a cubic anvil cell. **Figure** **1**a shows the X‐ray diffraction (XRD) patterns for each composition. In the range of 0 ≤ *x* ≤ 0.8, the main phase of the diffraction pattern could be indexed to the same simple K_2_NiF_4_‐type structure (space group: *I*4/*mmm*) as the reported high‐pressure phase (*x* = 0; *α*‐BLHO).^[^ ^5^ ^]^ Up to Na content *x* ≤ 0.6, a peak shift to lower angles with increasing *x* was detected (Figure 1b), and the calculated lattice constants increased linearly as a function of *x* following Vegard's law (Figure 1c). Given the larger ionic radius of Na^+^ (1.02 Å) compared to Li^+^ (0.76 Å),^[^ ^9^ ^]^ the observed lattice expansion indicates that the target solid solutions have been obtained. Rietveld analysis of the neutron diffraction (ND) pattern of *x* = 0.4 estimated a composition to be Ba_2_Li_0.645(3)_Na_0.355(3)_H_2.74(2)_O_1.160(4)_, which is close to the target Li/Na ratio. In contrast, for *x* = 0.8, impurity ratios increased significantly, and the lattice parameter is hardly changed from that of *x* = 0.6, suggesting that the solubility limit for Ba_2_Li_1–_ *_x_* Na*_x_* H_3_O lies in 0.6 < *x* < 0.8. Details of the refinement are given in supporting information (Figure S1 and Table S1, Supporting Information).

### 2.2 Phase Transition Behavior

**Figure 2:**
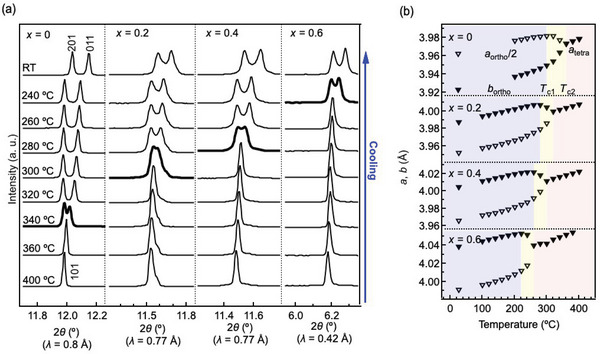
a) Temperature‐controlled SXRD profiles of Na‐BLHO synthesized by the high‐pressure reaction. Bold lines are the temperature corresponding to the phase transition at *T*_c2_. b) Temperature dependence of lattice constants for Na‐BLHO. The blue, yellow, and red highlighted areas are the *β*‐, *γ*‐, and *δ*‐phases estimated from the changes in lattice constants, respectively.

In order to investigate the phase transition of Na‐BLHO, temperature‐controlled synchrotron X‐ray diffraction (SXRD) measurements were performed for the sample of *x* = 0.2, 0.4, and 0.6 (Figure S2 a‐c, Supporting Information). As same with the reported phase change in BLHO (*x* = 0),^[^ ^5^ ^]^ the high‐pressure phase (*α*‐phase) with the simple K_2_NiF_4_‐type tetragonal cell irreversibly transformed to the ambient‐pressure phase (*β*‐ or *γ*‐phase) with the 2*a* × *b* × *c* orthorhombic supercell (space group: *Pnm*2_1_ or *Pnma*) with accompanying the extraction of BaO in the initial heating process. Subsequently, the reversible phase transition between the high‐temperature phase (*δ*‐phase) with the tetragonal cell (space group: *I*4/*mmm*) and the low‐temperature *β*‐ or *γ*‐phase underwent during the temperature rise to 400 °C and cooling to room temperature. Since the ambient‐pressure phases contribute to the ionic conductivity, here, we focused on the reversible phase transition after the initial heating. **Figure** **2**a shows the change in the (101) reflections with decreasing temperature as a typical peak. The temperature at which the split of 101 peak due to the phase change from tetragonal to orthorhombic (segregation point) was observed is lowered with increasing Na content *x*, suggesting stabilization of the *δ*‐phase by Na doping (*x* = 0, 340 °C; *x* = 0.2, 320 °C; *x* = 0.4, 300 °C; and *x* = 0.6, 260 °C). Change in lattice constant for each composition in the cooling process shows that the difference between the *a*‐ and *b*‐axes gradually widened over 20–30 °C from each segregation point, and then both parameters decreased linearly to room temperature with thermal shrinkage (Figure 2b). Assuming that Na‐BLHO has the same phase transition behavior as BLHO,^[^ ^5^ ^]^ these segregation and inflection points correspond to the temperatures at which the flipping of H_ap_/O_ap_ (from *δ*‐ to *γ*‐phase) and the order of H_eq_/*V*_H_ and Ba/*V*_Ba_ (from *γ*‐ to *β*‐phase) occur, respectively. Hereafter, these points are referred to as *T*_c1_ and *T*_c2_ from the low‐temperature side.

### 2.3 H^−^ Conductivity

**Figure 3:**
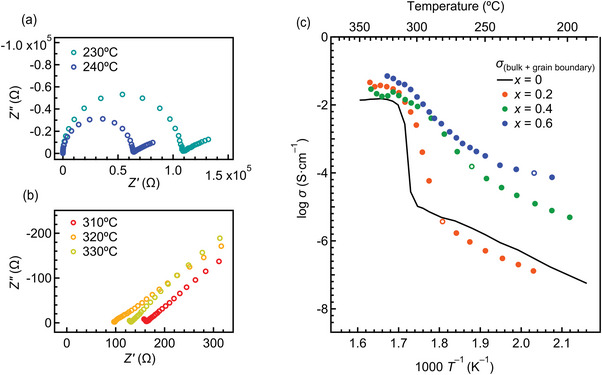
a–b) Cole‐Cole plots for the ambient‐pressure phase of Na‐BLHO (*x* = 0.4). c) Temperature dependencies of the total (bulk and grain boundary contributions) conductivity for each composition in Na‐BLHO. The unfilled circles are the conductivities at *T*_c1_, the *β*‐*γ* transition temperature estimated from the temperature‐controlled SXRD.

The ionic conductivities of *x* = 0.2, 0.4, and 0.6 were evaluated by the electrochemical AC impedance spectroscopy (EIS) in the temperature range 200 ≤ *T* ≤ 350 °C. Unlike BLHO (*x* = 0), the ambient‐pressure phase of Na‐BLHO cannot be synthesized directly, so the ambient‐pressure phase of each composition was prepared by annealing each sample obtained from the high‐pressure synthesis (Details in Figure S3, Supporting Information). The conductivity of Na‐BLHO could not be overestimated since the impurity phase (12–25 wt.%; BaO) generated during annealing is a typical insulator without ionic and electronic conductivities. **Figure** **3**a,b shows the collected impedance plots at 230, 240, 310, 320, and 330 °C for the composition of *x* = 0.4, as a typical example. Below 240 °C, the spectra show the specific signal of ion conductors consisting of a semicircle in the high‐frequency range and a spike in the low‐frequency range. These correspond to the sum of bulk and grain boundary resistances, and the electrode response, respectively. Above 310 °C, only a spike was observed due to the decrease in resistance (Figure 3b and S4, Supporting Information), and thus total conductivities based on bulk and grain boundary contributions at the high‐temperature region were estimated from the intercept of the spike. The temperature dependence of the conductivities for each composition is shown in Figure 3c, together with that of BLHO (*x* = 0).^[^ ^5^ ^]^ Discontinuous transitions to the high‐conductivity region deviating from the Arrhenius law were observed for each composition. The inflection points of the Arrhenius plots for each sample, which are the starting temperatures of the transition, approximately correspond to *T*_c1_ found in the change in lattice parameter (unfilled circles shown in Figure 3c) and shift to lower temperatures with increasing Na content. The conductivity of the tetragonal phase at high temperatures was over 10^−2^ S cm^−1^ for all compositions, but in the low‐temperature range (*T* < 300 °C), only the composition of *x* ≥ 0.4 showed an increase in the conductivity more than 100 times. Additionally, the slope on the Arrhenius plots from *T*_c1_ to the high‐conductivity region (*σ* ≥ 10^−2^ S cm^−1^) tends to become shallower with increasing Na content, implying that the transition behavior to the high conductive phase for Na‐BLHO was altered from BLHO. An ionic transport number at 320 °C for the sample of *x* = 0.4 was estimated to be 0.99 by the direct current (DC) polarization method, suggesting that electron (e^−^) and hole (h^+^) conductivities are negligible as a solid electrolyte (Figure S5, Supporting Information).

## 3 Discussion

**Figure 4:**
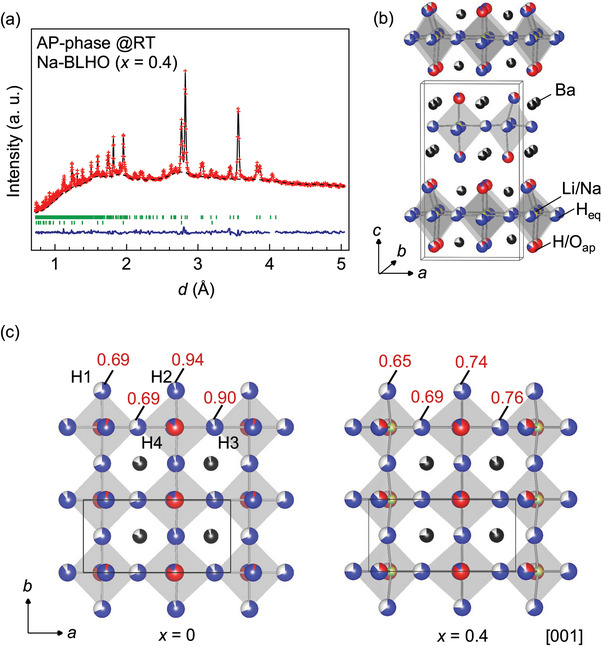
a) Rietveld refinement profile of ND data collected at RT for the ambient‐pressure (AP) phase of Na‐BLHO (*x* = 0.4). The final observed and calculated patterns are shown in red cross marks (+) and black solid lines, respectively. The blue solid line at the bottom of the plots is the residual difference from the fit to the observed data. The green tick marks correspond to the positions of Bragg reflections of the *Pnm*2_1_ orthorhombic phase (upper) and BaO (lower). b) Refined crystal structure of the AP‐phase of Na‐BLHO (*x* = 0.4). c) Crystal structures for BLHO (*x* = 0) and Na‐BLHO (*x* = 0.4) projected along the [001] direction.

To clarify the role of Na content on the temperature dependence of conductivities, the detailed crystal structures for the annealed samples of *x* = 0.2, and 0.4 were evaluated by ND. **Figure** **4**a shows the fitting profile of Rietveld refinement of ND data for *x* = 0.4 collected at room temperature. The determined structural parameters are summarized in **Table** **1**. The refinement result converges to a crystal structure based on the *β*‐BLHO with the three types of long‐range orderings (H_eq_/*V*_H_, H_ap_/O_ap_, and Ba/*V*_Ba_),^[^ ^5^ ^]^ which indicates that the same superlattice is maintained even in Na‐BLHO (Figure 4b). However, focusing on the occupancies of H_eq_ and Ba sites, the refined composition of Ba_1.60(3)_Li_0.6_Na_0.4_H_2.25(3)_O_0.959(8)_ has more vacancies than that of Ba_1.75_LiH_2.7_O_0.9_ (*x* = 0). Furthermore, as shown in Figure 4c, a contrast in the occupancy at Ba and equatorial anion site (*g*(Ba) = 0.74–0.85, and *g*(H_eq_) = 0.65–0.76) weakens, meaning that its crystal structure is closer to the high conductive *γ*‐phase in which the H_eq_/*V*_H_ and Ba/*V*_Ba_ are disordered. This is consistent with the fact that the conductivities in the low‐temperature range were enhanced for the compositions of *x* ≥ 0.4. Note that the rationale for the determination of the space group for *x* = 0.4 is described in the Supplemental text, Figure S7, and Tables S3–S4, Supporting Information. On the other hand, the composition of *x* = 0.2 had almost the same ordered structure as *x* = 0 (Figure S6a and Table S2, Supporting Information). The difference in the degree of ordering between *x* = 0.2 and 0.4 are thought to be mainly reflected in the conductivities at low temperature. The high‐temperature phase at *x* = 0.4 has the same highly vacant K_2_NiF_4_‐type structure (space group: *I*4/*mmm*) as *δ*‐BLHO,^[^ ^5^ ^]^ as confirmed by Rietveld refinement to ND data at 427 °C (Figure S8a and Table S5, Supporting Information).

**Table 1:** Refined structural parameters of the ambient‐pressure phase of Na‐BLHO (*x* = 0.4) at RT. Phase 1 (Na‐BHLO): 77.7 wt.%; Phase 2 (BaO): 22.3 wt.%

Refined composition: Ba_1.60(3)_Li_0.6_Na_0.4_H_2.25(3)_O_0.959(8)_						
Atom	Site	*g*	*x*	*y*	*z*	*B* / Å^2^
Ba1	2*a*	0.85(3)	0.1296(9)	0	0.8804(3)	0.39(4)
Ba2	2*a*	0.74(2)	0.66292 (9)	0	0.5933(3)	= *B*(Ba1)
Ba3	2*a*	0.85(2)	0.0997(7)	0	0.6107(3)	= *B*(Ba1)
Ba4	2*a*	0.75(3)	0.6060(9)	0	0.8857(4)	= *B*(Ba1)
Li/Na1	2*a*	0.6/0.4	0.152(6)	0	0.261(3)	1
Li/Na2	2*a*	0.6/0.4	0.633(11)	0	0.256(4)	1
H_eq_1	2*a*	0.65(2)	0.8731(15)	0	0.7584(6)	1.11(6)
H_eq_2	2*a*	0.74(3)	0.3634(11)	0	0.7387(6)	= *B*(H_eq_1)
H_eq_3	2*a*	0.76(3)	0.8949(10)	0	0.2494(5)	= *B*(H_eq_1)
H_eq_4	2*a*	0.69(3)	0.3595(12)	0	0.2530(5)	= *B*(H_eq_1)
H_ap_/O_ap_1	2*a*	0.605(6)/0.278(4)	0.071(3)	0	0.412(2)	0.43(15)
H_ap_/O_ap_2	2*a*	0.761 (9)/0.100(6)	0.6220(13)	0	0.0767(6)	= *B*(H_ap_1)
H_ap_/O_ap_3	2*a*	0.176(13)/0.662 (8)	0.1340(13)	0	0.0765(6)	0.20(5)
H_ap_/O_ap_4	2*a*	0.118 (18)/0.878(12)	0.6281(8)	0	0.4089(3)	= *B*(H_ap_3)

Space group *Pnm*2_1_, *a* = 8.02605(6) Å, *b* = 3.96559(3) Å, *c* = 14.24357(6) Å; *R*_wp_ = 0.651%, *R*_p_ = 0.477%, *S* = 8.37, *R*_B_ = 6.33%, *R*_F_ = 4.85%.

**Figure 5:**
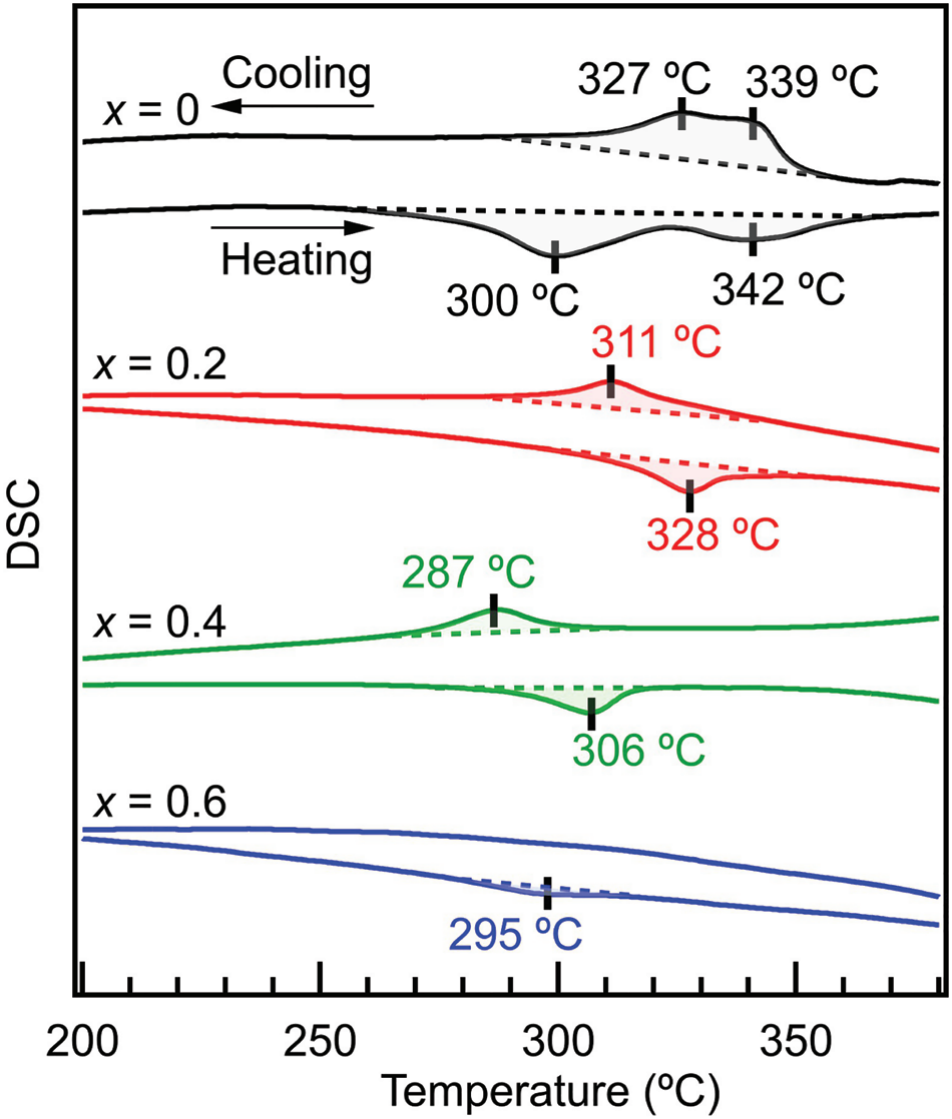
DSC scans of Na‐BLHO with the compositions of *x* = 0, 0.2, 0.4, 0.6.

The enthalpy change (Δ*H*_trans_) associated with the phase transition of Na‐BLHO was estimated by the differential scattering calorimetry (DSC) measurements. **Figure** **5** shows the DSC curves for each composition, together with that of BLHO.^[^ ^5^ ^]^ Unlike BLHO, where two pairs of endothermic and exothermic peaks corresponding to the *β*‐*γ* and *γ*‐*δ* transitions were observed at *T*_c1_ and *T*_c2_, respectively,^[^ ^5^ ^]^ the peak for Na‐BLHO was detected only around *T*_c2_, without a clear baseline shift. The Δ*H*_trans_ for each composition was calculated by integrating the endothermic peak areas and were summarized in **Table** **2**. The values of Δ*H*_trans_ decreased with increasing Na content and became so small at *x* = 0.6 that the peak could not be clearly identified, showing that the Na‐doping into BLHO has reduced the energy required to disorder the three types of long‐range ordering, H_eq_/*V*_H_, Ba/*V*_Ba_, and H_ap_/O_ap_. The fact that the endothermic (exothermic) peaks around *T*_c1_ were not detected in Na‐BLHO indicates that the Na‐doping more strongly affected the disorder of H_eq_/*V*_H_ and Ba/*V*_Ba_ corresponding to the *β*‐*γ* transition. The random distribution of Na and Li at the octahedral center may have facilitated the disordering, especially at the nearest neighbor H_eq_/V_H_. In addition to the DSC results, the slope of the Arrhenius plot during the transition to the high conductive phase becomes shallower with increasing Na content (Figure 3c), possibly suggesting that disorder of H_eq_/*V*_H_ and Ba/*V*_Ba_ occurs continuously in Na‐BLHO over a specific temperature range from *T*_c1_ to *T*_c2_.

**Table 2:** The phase transition enthalpy changes (∆*H*_trans_) were calculated from DSC curves for Na‐BLHO during the heating process. ∆*H*_trans_ for each composition is estimated in consideration of the amount of BaO as an impurity phase

Samples	*x* = 0	*x* = 0.2	*x* = 0.4	*x* = 0.6
∆*H_t_*_rans_ / kJ mol^−1^	1.86	1.20	0.78	0.45

## 4 Conclusion

In this study, we successfully synthesized the Ba–Li–Na–H‐O oxyhydride system (Na‐BLHO) with the K_2_NiF_4_‐type structure by the high‐pressure method. The Na‐BLHO, like a BLHO, has a tetragonal high‐pressure phase (*α*‐phase) and an orthorhombic ambient‐pressure phase with the 2*a* × *b* × *c* superlattice. The latter, which contributes to the ionic conductivity, was obtained only by annealing the high‐pressure phase. Detailed structural analysis by ND revealed that the ambient‐pressure phase at RT of Na‐BLHO is also the *β*‐phase with the three types of long‐range orderings, H_eq_/*V*_H_, Ba/*V*_Ba_, and H_ap_/O_ap_, but the degree of orderings for H_eq_/*V*_H_ and Ba/*V*_Ba_ reduced with increasing Na concentration, being closer to the structure of *γ*‐phase. The temperature dependence of the lattice parameters suggested that the *β*‐phase of Na‐BLHO has a similar phase change as BLHO in which the above‐mentioned long‐range orderings are successively disordered upon heating to form the *γ*‐, *δ*‐phases. With increasing the Na concentration, both temperatures of *T*_c1_ for the *β*‐*γ* transition and *T*_c2_ for the *γ*‐*δ* transition lowered, and the total enthalpy change required for the transition from the *β*‐ to the *δ*‐phase, i.e., the melt of three types of long‐range orderings, was reduced. The ionic conductivities of Na‐BLHO increased discontinuously from around *T*_c1_ according to the *β*‐*γ* transition and showed extremely high values over 10^−2^ S cm^−1^ for all compositions at temperatures above 300 °C. Furthermore, for *x* ≥ 0.4, the conductivity in the *β*‐phase is found to enhance more than two orders of magnitude in the low‐temperature range, which is attributed to a decrease in the degree of ordering for H_eq_/*V*_H_ and Ba/*V*_Ba_ at low temperature ran. Totally, the presented study reveals that Na‐doping into BLHO is effective for stabilizing the high conductive phase (*γ*‐, and *δ*‐BLHO). There remains room for further performance improvements, such as optimization of substituted species and their concentrations and multi‐element substitution, and thus stabilization of the high conductive phase of BLHO would be a principal guideline for developing hydride ion conductors.

## 5 Experimental Section

Powder samples of nominal composition Ba_2_Li_1−_ *_x_* Na*_x_* H_3_O (*x* = 0.2, 0.4, 0.6, 0.8) were synthesized by solid‐state reactions under high‐pressure using BaH_2_ (Mitsuwa Chemical, 99.5%), BaO (Aldrich, 99.99%), LiH (Aldrich, 95%) and NaH (Aldrich, 95%) as starting materials. A stoichiometric mixture of these materials was thoroughly ground and sealed in a BN sleeve covered with a NaCl capsule inside a pyrophyllite cell in an Ar‐filled glovebox. The cell was compressed to a pressure of 3 GPa using a cubic anvil press, heated at 650 °C for 1 h, and then quenched to room temperature, followed by a slow release of pressure. The ambient‐pressure phase was prepared by annealing the samples synthesized with a high‐pressure reaction in an H_2_ gas‐filled (0.42 MPa) stainless steel container at 350 °C for 6 h.

Laboratory powder XRD measurements were performed using a diffractometer (MiniFlex 600, Rigaku) with Cu‐K_*α*_ radiation. Since the samples were highly air‐sensitive, they were loaded into an airtight aluminum sample holder in the Ar‐filled glovebox. In an angle range of <15 °, peaks cannot be detected because of interference with the sample holder. Room temperature and temperature‐controlled powder SXRD measurements were performed at BL02B2^[^ ^10^ ^]^ at SPring‐8 in Japan. Diffraction data were collected in steps of 0.01° over a 2*θ* range of 3°–70°. The incident beam wavelength was calibrated using NIST SRM Ceria 640b CeO_2_, and then fixed at either 0.42 Å or 0.77 Å. The finely ground powder sample was loaded into a quartz capillary with an inner diameter of 0.3 mm. The sealed capillary was rotated during measurements to reduce the preferential orientation of the crystallites. Temperature‐controlled SXRD was measured in the range of 100–400 °C in 20 °C steps using the high‐temperature N_2_ gas flow system. Time‐of‐flite powder ND data for the samples loaded into a cylindrical vanadium cell (radius = 6 mm, height = 55 mm) were collected using SPICA and NOVA diffractometers at J‐PARC in Japan. The collected SXRD and ND profiles were analyzed by the Rietveld method using the RIETAN‐FP program^[^ ^11^ ^]^ for the SXRD data and the Z‐Rietveld program^[^ ^12^ ^]^ for the ND data. Crystal structures obtained by the refinements were illustrated by the VESTA program.^[^ ^13^ ^]^

DSC measurements were carried out using a Rigaku DSC 8231 instrument. Powder samples of Na‐BLHO (*x* = 0.2, 0.4, 0.6) after annealing were placed in a crimped Al pan and scanned heat fluxes during the heating and cooling processes in the range from room temperature to 400 °C at a rate of 10 °C min^−1^. The samples were purged with Ar gas throughout the measurements.

Ionic conductivity of the Na‐BLHO after annealing were measured by EIS under H_2_ gas flow at 200 °C ≤ *T* ≤ 350 °C, with an applied frequency range from 0.1 Hz to 35 MHz, using Bio‐Logic MTZ‐35 frequency response analyzer. The ion‐blocking Mo electrode was deposited on both sides of the sintered pellet samples with a diameter of ≈4.0 mm and a thickness of ≈1.3 mm. The obtained impedance spectra were fitted with electrical equivalent circuits using the EC‐Lab software. The DC electronic conductivity of *x* = 0.4 sample of Na‐BLHO was measured using a symmetric Mo/sample/Mo cell with an applied voltage of 300 mV at 320 °C.

## Conflict of Interest

The authors declare no conflict of interest.

## Author Contributions

G.K. conceived, supervised, and designed the whole study. K.O. carried out the whole experiments, including material synthesis, electrochemical measurements, structural refinement of XRD and ND, and thermal analysis, under the supervision of F.T and G.K. Y.I. optimized synthesis conditions. M.Y., T.S., K.I., T.O., and T.K. collected ND data. All authors discussed the results; K.O., F.T., and G.K. wrote the manuscript.

## Supporting information

Supporting Information

## Data Availability

The data that support the findings of this study are available from the corresponding author upon reasonable request.
