# Supplementary material for: Impact of Na Concentration on the Phase Transition Behavior and H− Conductivities in the Ba–Li–Na–H–O Oxyhydride System
Source: Adv Sci (Weinh). 2022 Nov 16;10(1):2203541. doi: 10.1002/advs.202203541 (PMC9811434; doi:10.1002/advs.202203541)
Supplement: Supplementary file 1 — Supporting Information [file ADVS-10-2203541-s001.pdf]

ADVANCED SCIENCE
Open Access

Supporting Information

for *Adv. Sci.*, DOI 10.1002/advs.202203541

Impact of Na Concentration on the Phase Transition Behavior and H^- Conductivities in the Ba–Li–Na–H–O Oxyhydride System

*Kei Okamoto, Fumitaka Takeiri, Yumiko Imai, Masao Yonemura, Takashi Saito, Kazutaka Ikeda, Toshiya Otomo, Takashi Kamiyama and Genki Kobayashi**

Supporting Information

Impact of Na Concentration on the Phase Transition Behavior and H^{-} Conductivities in the Ba-Li-Na-H-O oxyhydride system

Kei Okamoto, Fumitaka Takeiri, Yumiko Imai, Masao Yonemura, Takashi Saito, Kazutaka Ikeda, Toshiya Otomo, Takashi Kamiyama, and Genki Kobayashi*

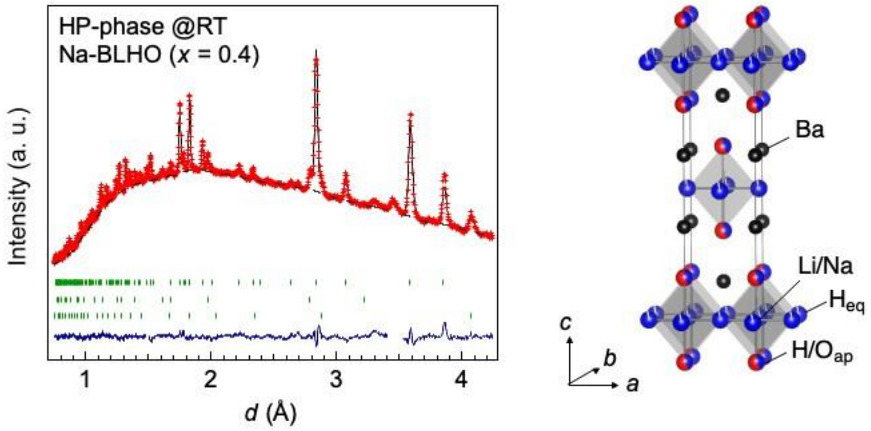

Figure S1. Rietveld refinement profile of ND data collected at RT for Na-BLHO (x =0.4) synthesized by high-pressure (HP) reaction. Final observed and calculated patterns are shown in red cross marks and black solid line, respectively. The blue solid line at the bottom of the plots is residual difference from the fit to the observed data. The green tick marks correspond to the positions of Bragg reflections of *I*4/*mmm* tetragonal phase (upper), BaO (middle), and $BaLiH_3$ (lower), respectively. Right figure is a refined crystal structure.

Table S1. Refined room-temperature structural parameters of high-pressure phase of Na-BLHO (x = 0.4) at room temperature.

Phase 1: $Ba_2Li_{0.645(3)}Na_{0.355(3)}H_{2.74(2)}O_{1.160(4)}$ (90.7 wt%)

Atom	Site	g	x	y	z	B / Å^2
Ba	4*e*	1	0	0	0.36076(10)	0.86(7)
Li/Na	2*a*	0.645(3) / 1–g(Li)	0	0	0	1
H_{eq}	4*c*	0.925(11)	0	0.5	0	1.75(11)
H_{ap}/O_{ap}	4*e*	0.420(2) / 1–$g(O_{ap})$	0	0	0.1639(3)	1.15(16)

Space group *I*4/*mmm*, a = 3.97234(6) Å, c = 14.2225(5) Å;
R_{wp} = 1.16%, R_p = 0.925%, S = 1.95, R_B = 9.29%, R_F = 6.95%.

Phase 2: BaO (6.8 wt%)

Atom	Site	g	x	y	z	B / Å^2
Ba	4*a*	1	0	0	0	0.5
O	4*b*	1	0.5	0.5	0.5	1

Space group *Fm*–3*m*, a = 5.5267(4) Å

Phase 3: $BaLiH_3$ (2.5 wt%)

Atom	Site	g	x	y	z	B / Å^2
Ba	1*b*	1	0.5	0.5	0.5	0.5
Li	1*a*	1	0	0	0	1
H	3*d*	1	0.5	0	0	1

Space group *Pm*–3*m*, a = 4.0364(7) Å

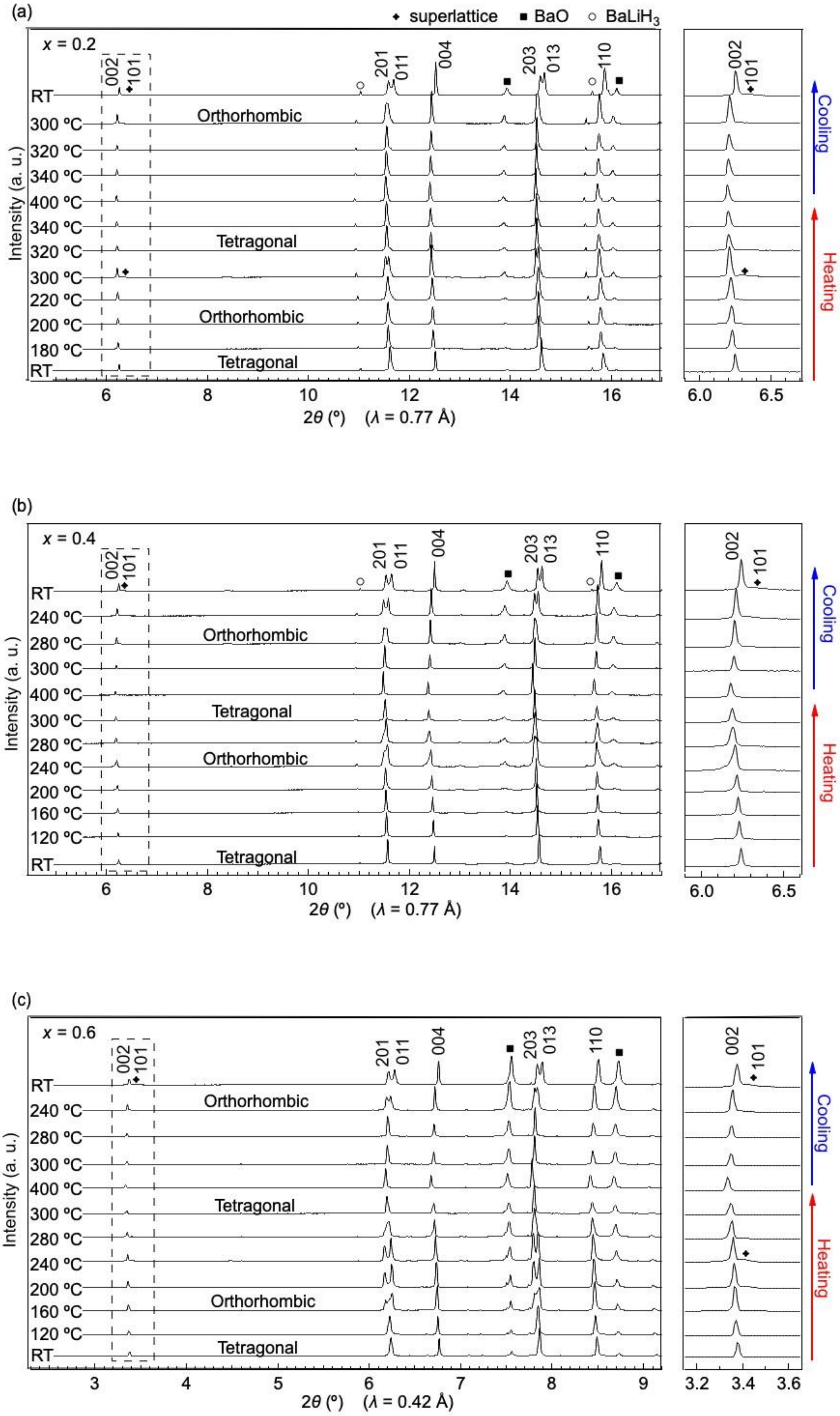
(a)
◆ superlattice ■ BaO ○ BaLiH3
x = 0.2
002
101
201
011
004
203
013
110
RT
300 ºC
320 ºC
340 ºC
400 ºC
340 ºC
320 ºC
300 ºC
220 ºC
200 ºC
180 ºC
RT
Orthorhombic
Tetragonal
Orthorhombic
Tetragonal
Intensity (a. u.)
2θ (º) (λ = 0.77 Å)
Cooling
Heating
(b)
x = 0.4
RT
240 ºC
280 ºC
300 ºC
400 ºC
300 ºC
280 ºC
240 ºC
200 ºC
160 ºC
120 ºC
RT
Orthorhombic
Tetragonal
Orthorhombic
Tetragonal
2θ (º) (λ = 0.77 Å)
(c)
x = 0.6
RT
240 ºC
280 ºC
300 ºC
400 ºC
300 ºC
280 ºC
240 ºC
200 ºC
160 ºC
120 ºC
RT
Orthorhombic
Tetragonal
Orthorhombic
Tetragonal
2θ (º) (λ = 0.42 Å)

Figure S2. Temperature-controlled SXRD patterns for the samples of x = (a) 0.2, (b) 0.4, and (c) 0.6.

Preparation of the ambient-pressure phase of Na-BLHO

Direct synthesis of Na-BLHO by solid-state reaction under ambient pressure was impossible because the raw material, NaH, tends to volatilize during sintering. Therefore, we prepared the ambient-pressure phase of Na-BLHO by annealing the sample synthesized with a high-pressure reaction under H_2 atmosphere. Figure S3 (a) – (b) show the X-ray diffraction patterns for each composition before and after annealing. In all compositions, lowering symmetry from tetragonal to orthorhombic was found after the annealing, and a slight increase in the amount of impurity (BaO) was observed. The change in the lattice parameter according to Vegard's law was confirmed even after annealing, indicating that the Na-concentration was maintained in the ambient-pressure phase (Fig. S3 (c)).

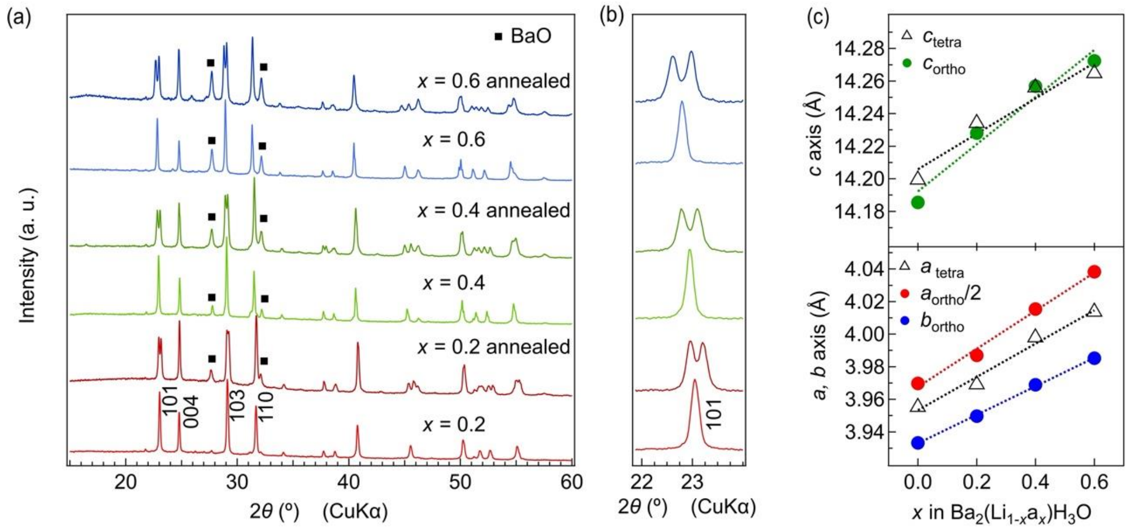

Figure S3. (a) XRD patterns of Na-BLHO before and after annealing. BaO is extracted by annealing. (b) Enlarged XRD patterns of (101) reflection. (c) Lattice constants estimated from Rietveld refinement analysis to XRD date.

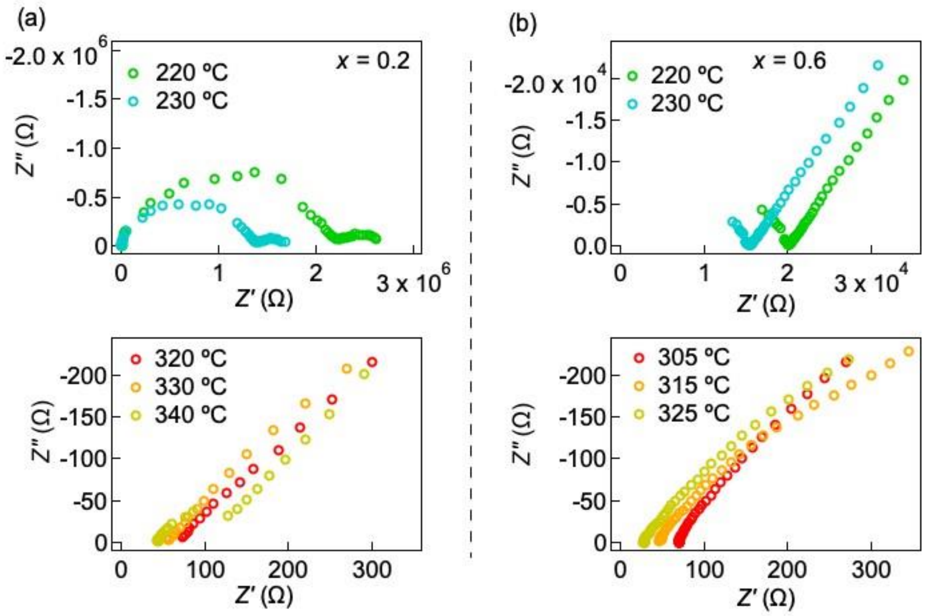

Figure S4. Cole-Cole plots for the composition of (a) x = 0.2 and (b) 0.6 in Na-BLHO.

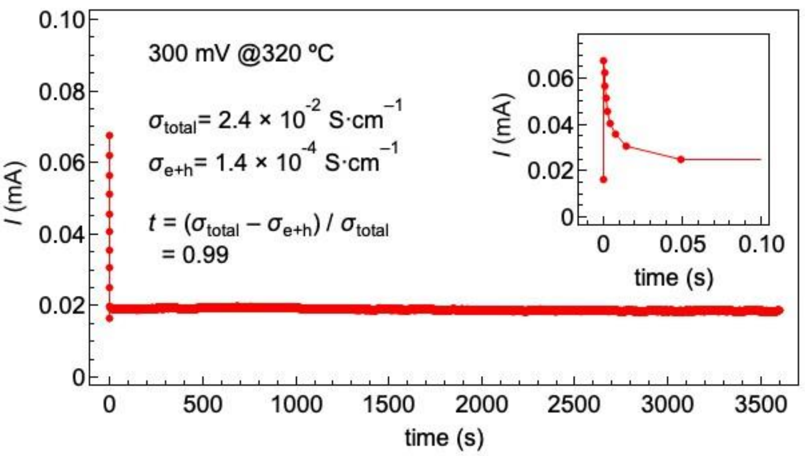

Figure S5. DC polarization curve obtained from Mo|Na-BLHO (x = 0.4)|Mo symmetric cell at 320 ºC.

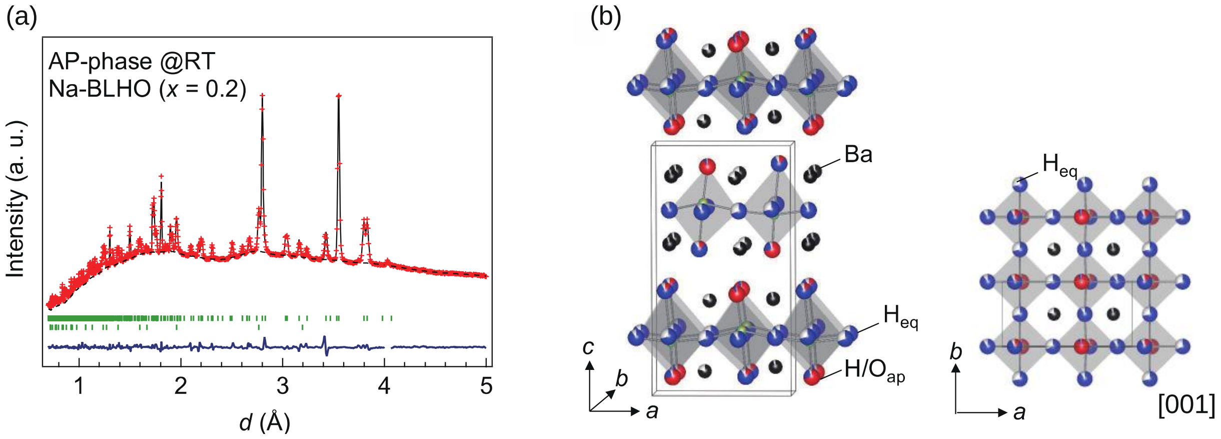

Figure S6. (a) Rietveld refinement profile of ND data collected at RT for the ambient-pressure (AP) phase of Na-BLHO (x = 0.2). The final observed and calculated patterns are shown in red cross marks and black solid line, respectively. The blue solid line at the bottom of the plots is the residual difference from the fit to the observed data. The green tick marks correspond to the positions of Bragg reflections of the $Pnm2_1$ orthorhombic phase (upper) and BaO (lower). (b) Determined crystal structure for x = 0.2.

Table S2. Refined structural parameters for the ambient-pressure phase of Na-BLHO (x = 0.2) at RT.

Phase 1: $Ba_{1.734(5)}Li_{0.8}Na_{0.2}H_{2.495(5)}O_{0.962(2)}$ (90.6wt%)

Atom	Site	*g*	*x*	*y*	*z*	B / Å^2
Ba1	2*a*	0.941(4)	0.14367(18)	0	0.87823(7)	0.317(11)
Ba2	2*a*	0.802(5)	0.6369(2)	0	0.58884(9)	= *B*(Ba1)
Ba3	2*a*	0.933(5)	0.12812(18)	0	0.60644(7)	= *B*(Ba1)
Ba4	2*a*	0.791(4)	0.60063(19)	0	0.87119(9)	= *B*(Ba1)
Li/Na1	2*a*	0.8 / 0.2	0.1346(13)	0	0.2146(7)	1
Li/Na2	2*a*	0.8 / 0.2	0.6455(10)	0	0.2612(7)	1
$H_{eq}1$	2*a*	0.711(4)	0.8631(3)	0	0.7465(2)	1.05(2)
$H_{eq}2$	2*a*	0.887(5)	0.3633(3)	0	0.7358(2)	= $B(H_{eq}1)$
$H_{eq}3$	2*a*	0.891(4)	0.8875(3)	0	0.232261(19)	= $B(H_{eq}1)$
$H_{eq}4$	2*a*	0.696(4)	0.3896(4)	0	0.2295(2)	= $B(H_{eq}1)$
$H_{ap}/O_{ap}1$	2*a*	0.7618(18) / 0.1789(12)	0.0972(4)	0	0.415(2)	0.59(4)
$H_{ap}/O_{ap}2$	2*a*	0.754(2) / 0.1403(16)	0.6740(4)	0	0.0776(2)	= $B(H_{ap}1)$
$H_{ap}/O_{ap}3$	2*a*	0.210(2) / 0.7247(14)	0.14920(18)	0	0.07522(17)	0.358(17)
$H_{ap}/O_{ap}4$	2*a*	0.068(3) / 0.912(2)	0.61267(18)	0	0.40664(10)	= $B(H_{ap}3)$

Space group $Pnm2_1$, a = 7.95680(2) Å, b = 3.940349(10) Å, c = 14.19220(5) Å;
R_{wp} = 0.981%, R_p = 0.627%, S = 19.8, R_B = 5.48%, R_F = 4.80%.

Phase 2: BaO (9.4wt%)

Atom	Site	*g*	*x*	*y*	*z*	B / Å^2
Ba	4*a*	1	0	0	0	0.5
O	4*b*	1	0.5	0.5	0.5	1

Space group *Fm*–3*m*, a = 5.53200(2) Å

The structural refinements for Na-BLHO (*x* = 0.4) at room temperature

To determine the crystal structure of Na-BLHO (x = 0.4), we performed Rietveld analyses using structural models of both β- and γ-phases. Figure S7 (a) shows the fitting profile of Rietveld refinement of ND data using the structural model of γ-BLHO with *Pnma* space group. The determined structural parameters are summarized in Table S3. Similar to the refinement results using the structural model of the β-phase with space group $Pnm2_1$ shown in Figure 4 and Table 1, the calculated pattern is an acceptable fit for the observed pattern, but the R-factors (Table S4) showed slightly higher reliability values for the β-phase model. The crystal structure of the β-phase with space group $Pnm2_1$ belongs to the subgroup of the γ-phase with space group *Pnma*, and both Ba and H_{eq} sites increase from two to four due to ordering V_{Ba} and V_H. In our previous work on the phase transition of BLHO[5], we discriminated the β- and γ-phase by whether the vacancies introduced at the Ba and H_{eq} sites

are ordered or not. Based on this previous finding and the result that refinement using the β-phase model (shown in Fig. 4 and Table 1) confirmed the contrast in occupancies of the Ba and equatorial H sites, respectively, we concluded that the sample of x = 0.4 retains the low symmetric β-phase.

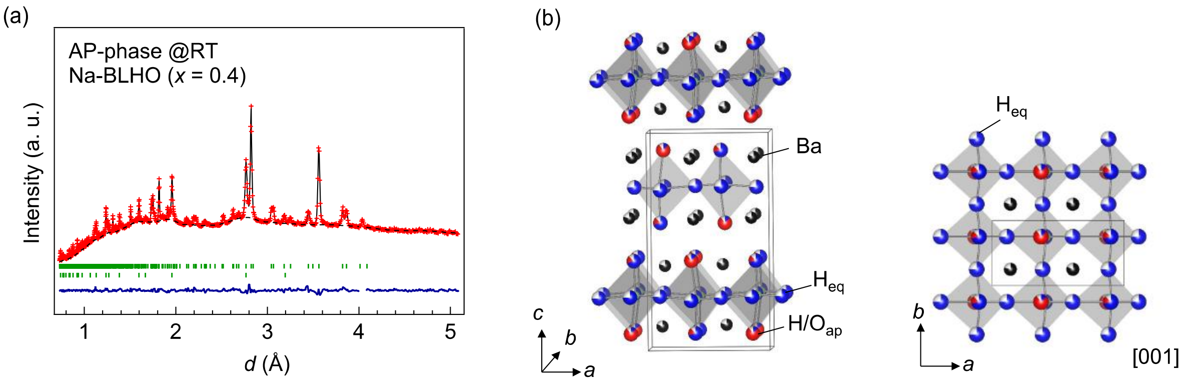

Figure S7. (a) Rietveld refinement profile of ND data collected at RT for the ambient-pressure (AP) phase of Na-BLHO (x = 0.4). The final observed and calculated patterns are shown in red cross marks and black solid lines, respectively. The blue solid line at the bottom of the plots is the residual difference from the fit to the observed data. The green tick marks correspond to the positions of Bragg reflections of *the Pnma* orthorhombic phase (upper) and BaO (lower). (b) Determined crystal structure for x = 0.4.

Table S3. Refined structural parameters for the ambient-pressure phase of Na-BLHO (x = 0.4) at RT using the model of γ-phase.

$Ba_{1.61(2)}Li_{0.6}Na_{0.4}H_{2.22(2)}O_{0.931(7)}$

Atom	Site	*g*	*x*	*y*	*z*	*B* / Å^2
Ba1	4*c*	0.819(17)	0.1107(3)	0.25	0.10770(8)	0.49(3)
Ba2	4*c*	0.789(16)	0.1364(3)	0.25	0.38608(9)	*B*(Ba1)
Li/Na1	4c	0.6 / 0.4	0.408(3)	0.75	0.2404(14)	1
$H_{eq}1$	4c	0.746(16)	0.3808(6)	0.25	0.24152(14)	1.21(4)
$H_{eq}2$	4c	0.756(16)	0.1089(5)	0.75	0.25308(14)	$B(H_{eq}1)$
$H_{ap}/O_{ap}1$	4c	0.715(5) / 0.168(3)	0.3978(6)	0.75	0.0835(2)	0.62(12)
$H_{ap}/O_{ap}2$	4c	0.119(11) / 0.763(7)	0.3691(3)	0.75	0.41777(9)	0.36(5)

Space group *Pnma*, a = 8.02627(6) Å, b = 3.96556(3) Å, c = 14.24363(5) Å;
R_{wp} = 0.693%, R_p = 0.505%, S = 8.80, R_B = 8.68%, R_F = 6.52%.

Table S4. Comparison of R-factors between refinements using model of β- and γ-phases of Na-BLHO (x = 0.4) at RT.

	GoF	R_{wp}	R_p	R_B	R_F
β-phase model	8.37	0.651	0.477	6.33	4.85
γ-phase model	8.80	0.693	0.505	8.68	6.52

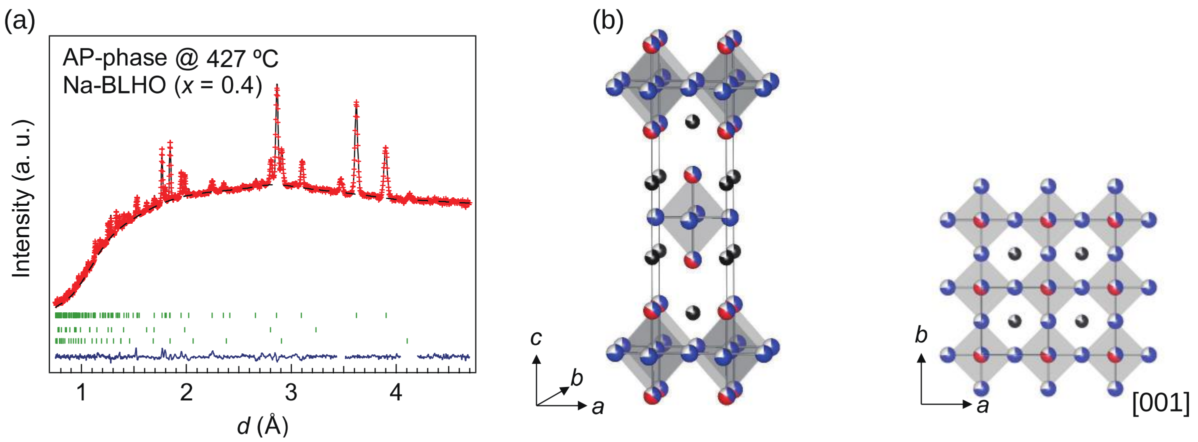

Figure S8. (a) Rietveld refinement profile of ND data collected at 427 ºC for the ambient-pressure (AP) phase of Na-BLHO (x = 0.4). Final observed and calculated patterns are shown in red cross marks and black solid line, respectively. The blue solid line at the bottom of the plots is residual difference from the fit to the observed data. The green tick marks correspond to the positions of Bragg reflections of *I*4/*mmm* tetragonal phase (upper), BaO (middle), and $BaLiH_3$ (lower). (b) Determined crystal structure for x = 0.4.

Table S5. Refined structural parameters for the ambient-pressure phase of Na-BLHO (x = 0.4) at 427 ºC.

Phase 1: $Ba_{1.59}Li_{0.6}Na_{0.4}H_{2.23(2)}O_{0.922(8)}$ (91.8wt%)

Atom	Site	g	x	y	z	B / Å^2
Ba	4*e*	0.795	0	0	0.35991(14)	1.05(10)
Li/Na	2*a*	0.6 / 0.4	0	0	0	1
H_{eq}	4*c*	0.741(10)	0	0.5	0	1.92(15)
H_{ap}/O_{ap}	4*e*	0.372(7) / 0.461(4)	0	0	0.1603(4)	1.1(2)

Space group *I*4/*mmm*, a = 4.01026(8) Å, c = 14.3485(7) Å;
R_{wp} = 1.20%, R_p = 0.878%, S = 1.89, R_B = 7.57%, R_F = 7.81%.

Phase 2: BaO (7.1wt%)

Atom	Site	g	x	y	z	B / Å^2
Ba	4*a*	1	0	0	0	0.5
O	4*b*	1	0.5	0.5	0.5	1

Space group *Fm*–3*m*, a = 5.5605(3) Å

Phase 3: $BaLiH_3$ (1.1wt%)

Atom	Site	g	x	y	z	B / Å^2
Ba	1*b*	1	0.5	0.5	0.5	0.5
Li	1*a*	1	0	0	0	1
H	3*d*	1	0.5	0	0	1

Space group *Pm*–3*m*, a = 4.0878(10) Å
